# Disentangling the contribution of inflammatory markers and kynurenines to cognitive impairment in schizophrenia

**DOI:** 10.1192/j.eurpsy.2025.419

**Published:** 2025-08-26

**Authors:** J. Sapienza, G. Agostoni, S. El Sagher, M. D’Incalci, M. Spangaro, M. Bechi, M. Buonocore, F. Cocchi, S. Comai, R. Cavallaro, M. Bosia

**Affiliations:** 1 Neurosciences, IRCSS San Raffaele, Milan; 2 Humanities and Life Sciences, IUSS, Pavia; 3 Vita-Salute San Raffaele University, Milan, Italy; 4 Neurosciences, Vita-Salute San Raffaele University, Milan; 5 Department of Pharmaceutical and Pharmacological Sciences, University of Padua, Padua, Italy

## Abstract

**Introduction:**

Neuroinflammation and the Kynurenine Pathway (KP) have gained attention in the last decades in the pathogenesis of cognitive impairment in schizophrenia. Pro-inflammatory cytokines and microglia activation induce oxidative stress, neurodegeneration, white matter (WM) disruption and increased synaptic pruning and, importantly, activate the KP, whose metabolites have neurotoxic/neuroprotective and neuromodulatory properties on cholinergic and glutamatergic neurotransmission, two pivotal systems in cognitive processes.

**Objectives:**

This study aims to investigate the relationship between levels of inflammatory markers and KP metabolites and cognition in schizophrenia with a focus on the differential impact of these biomarkers on the different phases of the illness.

**Methods:**

Associations between levels of biomarkers and cognitive domains in the wholw sample of 120 patients with schizophrenia were firstly assessed. Then, patients were divided in two subsamples depending on the duration of illness, with the aim to evaluate the impact of inflammatory biomarkers and kynurenines on cognition depending on disease progression. Finally, we performed cluster analysis to investigate kynurenines as possible clustering variables with the final aim to attribute a different cognitive profile to each cluster.

**Results:**

In the whole sample we found negative correlations between multiple inflammatory markers including IL-1β, IL-6, TNF-α, and cognitive functions, particularly verbal memory. Negative associations between verbal memory and TNF-a, IFNg and IL-5 were found in early-phase patients compared to late-phase patients, who showed a less strong associations. Interestingly, kynurenines showed significant associations with cognition in multiple areas regardless of the duration of illness. Regarding clustering, Cluster 1 included patients with lower levels of Tryptophan, Quinolinic Acid, and Kynurenic Acid, as well as higher levels of 3-hydroxykynurenine, compared to Cluster 2 (Fig. 1). Interestingly the two clusters showed different cognitive profiles. Verbal memory, psychomotor speed and attention significantly differed between the two clusters, with Cluster 1 showing the most impaired cognition in all these domains (Fig. 2).

**Image 1:**

**Figure:**
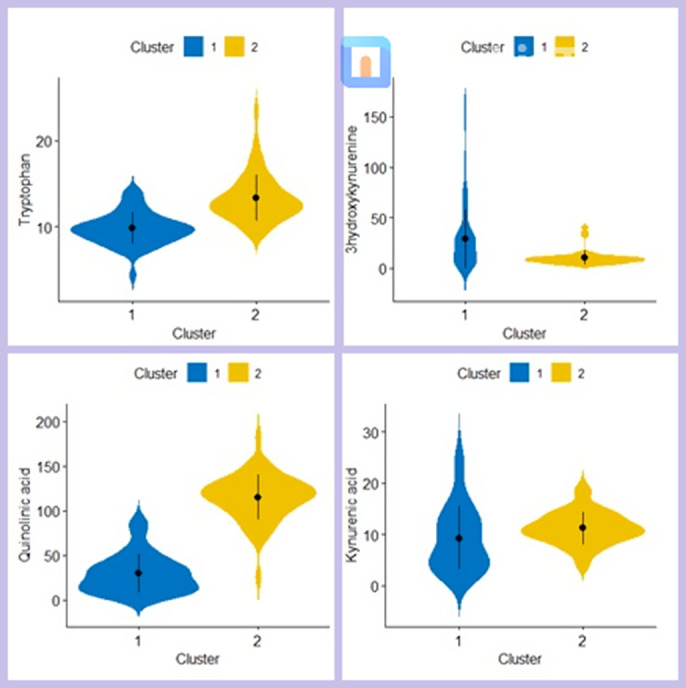

**Image 2:**

**Figure:**
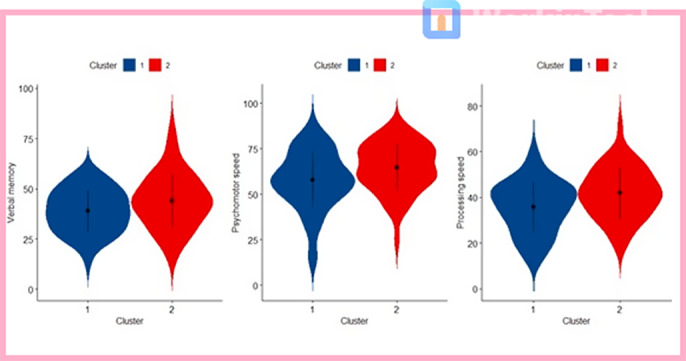

**Conclusions:**

These results stress the primary importance of inflammation and KP abnormalities in cognitive impairment. The effects of inflammation on cognition seems to decline over time, while metabolites of the kynurenine pathway continue to have an impact. Probably, pro-inflammatory cytokines impact cognition more in patients with a shorter duration of illness as the biological bases of cognitive functions are more preserved (cortical volumes, synapses, WM integrity), whilw the neuromodulation of KP metabolites combined with their neurotoxic/neuroprotective profile can explain the diferential effect.

**Disclosure of Interest:**

None Declared

